# Cellulose-mediated floc formation by the activated sludge bacterium *Shinella zoogloeoides* ATCC 19623

**DOI:** 10.1186/s12866-022-02516-y

**Published:** 2022-04-15

**Authors:** Na Gao, Jingcheng Dai, Yaqi Liu, Shuyang Li, Jing Wang, Wenxuan Lu, Dongru Qiu

**Affiliations:** 1 Institute of Hydrobiology, Chinese Academy of Sciences, Wuhan, 430072 China; 2 Key Laboratory of Freshwater Aquaculture and Enhancement of Anhui Province, Fisheries Research Institute, Anhui Academy of Agricultural Sciences, Hefei, 230031 China; 3 University of Chinese Academy of Sciences, Beijing, 100049 China

**Keywords:** Cellulose, floc formation, *Shinella zoogloeoides*, activated sludge

## Abstract

**Background:**

Bacterial floc formation plays a central role in the activated sludge (AS) process. The formation of AS flocs has long been known to require exopolysaccharide biosynthesis. We had demonstrated that both expolysaccharides and PEP-CTERM (a short C-terminal domain includes a near-invariant motif Pro-Glu-Pro (PEP)) proteins were required for floc-forming in *Zoogloea resiniphila* MMB, a dominant AS bacterium. However, the PEP-CTERM proteins are not encoded in the genome of AS bacterium *Shinella zoogloeoides* ATCC 19623 (formerly known as *Zoogloea ramigera* I-16-M) and other sequenced AS bacteria strains. The mechanism underlying floc formation of *Shinella* and related AS bacteria remained largely unclear.

**Results:**

In this study, we have sequenced and annotated the complete genome of *S. zoogloeoides* ATCC 19623 (*aka* I-16-M), previously isolated in USA and treated as the neotype for the AS floc-forming bacterium *Zoogloea ramigera* I-16-M, and another AS strain XJ20 isolated in China. *Mariner* transposon mutagenesis had been conducted to isolate floc-forming-deficient mutants in the strain ATCC 19623 as previously performed by using *Tn5* transposon three decades ago. The transposon insertional sites of multiple mutants were mapped to the gene cluster for bacterial cellulose synthesis (*bcs*) and secretion, and the role played by these genes in floc-formation had been further confirmed by genetic complementation. Interestingly, the restriction map of this *bcs* locus-flanking region was highly similar to that of the previously identified DNA fragment required for floc-formation in 1980s. Cellulase treatment abolished the floc-forming phenotype of *S. zoogloeoides* ATCC 19623 but not that of *Z*. *resiniphila* MMB strain. The FTIR spectral analyses revealed that the samples extracted from *S. zoogloeoides* ATCC 19623 were cellulose polymer.

**Conclusion:**

Our results indicated that we have largely reproduced and completed the unfinished pioneering work on AS floc-formation mechanism, demonstrating that the floc-formation and flocculating capability of *Shinella* were mediated by extracellular cellulose polymers.

**Supplementary Information:**

The online version contains supplementary material available at 10.1186/s12866-022-02516-y.

## Background

The AS process has been widely applied for municipal sewage and industrial wastewater treatment worldwide and has played a central role in water purification for over a century. *Zoogloea ramigera* and related floc-forming bacteria are required for formation of AS flocs key to gravitational effluent-and-sludge separation and AS recycling, two key elements for AS process. Butterfield *et al*. isolated a floc-forming organism from activated sludge and tentatively identified it as *Z. ramigera*, based on the phenotypes that the bacterial culture was capable of forming flocs similar to normal activated sludge and also exhibited oxidation characteristics similar to those of normal activated sludge [1]. Bacteria of the genus *Zoogloea* have historically been differentiated from other obligately aerobic, Gram-negative, non-spore-forming, rod-shaped bacteria that grow in aquatic habitats on the basis of a characteristic gelatinous matrix, often called a “zoogloeal matrix”, that surrounds clumps of cells when they are found in their aquatic habitat or in unshaken liquid culture in the laboratory [2]. However, the specimen had not been preserved and the taxonomy and microbiology of the *Z. ramigera* species were incomplete without a type strain. Therefore, three neotype strains for floc-forming *Z. ramigera* had been isolated from activated sludge and experimentally characterized during the 1960s [3–5]. However, these three strains obviously did not belong to the same species and one strain, ATCC 19544 (*aka* N.C. Dondero 106 strain), directly isolated by microdissection of a natural branched zoogloeal floc [5], has been recognized taxonomically as the neotype strain for the species of *Z. ramigera* Itzigsohn [6, 7]. The other two floc-forming strains, ATCC 19623 (*aka* I-16-M) and ATCC 25935 (*aka* P.R. Dugan 115 strain), were reclassified as novel species *S. zoogloeoides* of *Rhizobiales* and *Duganella zoogloeoides* of *Burkholderiales*, respectively, based on the phenotypic and 16S rDNA-based phylogenetic analyses [8–10]. In addition, another name *Crabtreella saccharophila* gen. nov., sp. nov. was proposed for strain ATCC 19623 (*aka* I-16-M) based on phylogenetic and chemotaxonomic analyses in March, 2006 [11]. Because *S. zoogloeoides* was proposed before *Crabtreella saccharophila*, the latter name is not a valid name according to the rules of *the International Code of Nomenclature of Bacteria.* All three could flocculate and each strain produces a different extracellular polysaccharide [12]. *S. zoogloeoides* ATCC 19623 strain produces a cellulose fibrillar polysaccharide [12], *Z. ramigera* ATCC 19544 (*aka* 106) produces an exopolymer reportedly containing amino sugars [13], and *Duganella zoogloeoides* ATCC 25935 (*aka* 115) produces a weakly acidic extracellular polysaccharide composed of D-glucose, D-galactose and pyruvic acid in an approximate molar ratio 11:3:1.5 [14, 15]. Anthony J. Sinskey *et al.* pioneered the study of genetics and molecular mechanism underlying the bacterial floc formation using *S. zoogloeoides* ATCC 19623 (still classified as *Z. ramigera* I-16-M at that time) as a model strain [16]. By using *Tn5* transposon mutagenesis, they isolated the *Tn5* insertion mutants deficient in EPS production by screening for the absence of fluorescence on plates containing the dye Calcofluor. They further determined whether a specific chromosomal DNA segment was required for exopolysaccharide biosynthesis and floc formation based on the planktonic cell growth (turbid cell culture) of the mutant and recovery of floc-forming phenotype and gravitational settling property of the mutants by genetic complementation using a genetic library, constructed in a broad-host-range cosmid vector, and introduced into the mutants via conjugation [16]. However, due to lack of easy sequencing methods in those days, the relevant genes had not been identified and the chromosomal DNA inserts of complementing recombinant plasmids had not been sequenced. In recent years, we have employed the similar methodology and techniques to identify the genes and gene clusters required for *Z. resiniphila* and other predominant floc-forming bacteria [17–19]. Our research has been greatly facilitated by high-throughput genome sequencing and more powerful genetic manipulation tools. Our previous researches had revealed that the formation of AS flocs requires both exopolysaccharide biosynthesis and expression of the PEP-CTERM domain proteins in *Z. resiniphila*, a dominant AS bacterium [17, 19]. The PEP-CTERM protein-sorting domain is usually a 25-residue carboxyl terminal domain that includes a near-invariant Proline-Glutamate-Proline (PEP) motif, a thirteen residue strongly hydrophobic sequence likely to span the membrane, and a five-residue strongly basic motif that often contains four arginine residues [20]. This kind of proteins also contain an amino terminal secretion signal peptide and the asparagine (N) residue is overrepresented and could be used for N-linked glycosylation [20]. A member of a wide-spread family of high copy number-per-genome PEP-CTERM genes were transcriptionally regulated by the RpoN (sigma^54^) sigma factor and PrsK-PrsR two-component system. Without PrsK or PrsR, *Z. resiniphila* cells were planktonic rather than aggregated into the floc structures that allow gravitational sludge settling and recycling. Moreover, the biosynthesis and secretion of exopolysaccharides remained unaffected in the prsK and prsR mutants. But the synthesized exopolysaccharides were released into the growth broth in soluble form. Overexpression of PEP-CTERM A protein (PepA) could circumvent the requirement of PrsK and PrsR for floc-formation [19]. We had also demonstrated that one of the four RpoN paralogues, RpoN1, regulated the floc formation but not exopolysaccharide biosynthesis in a floc-forming *Aquincola tertiaricarbonis* RN12 strain [18] and *Azoarcus halotolerans* [21]. The synthesized exopolysaccharide chains were not tightly bound to the bulk of cell aggregates but were released into the cultivation broth. A highly conserved large gene cluster, including two asparagine synthetase genes and other genes involved in exopolysaccharide biosynthesis and secretion, was encoded in the genome of *Z. resiniphila* MMB and *A. tertiaricarbonis* RN12 [17, 18]. *Interestingly, both* PEP-CTERM proteins and exopolysaccharide biosynthesis pathway were also encoded in the genome of many other AS bacteria including comammox strains of *Nitrospira* and polyphosphate-accumulating organisms *Candidatus* Accumulibacter phosphatis [17, 19].

Since a series of researches such as the biosynthesis of polyhydrobutyrate (PHB), one of the bioplastic materials, have also been intensively conducted in *S. zoogloeoides* ATCC 19623 [22], but the genome data of *S. zoogloeoides* ATCC 19623 has not yet been published at the time of writing. In the present study, we sequenced and annotated the complete genome of the two *S. zoogloeoides* strains and conducted comparative genomics and molecular genetics analyses on the floc formation using *mariner* transposon mutagenesis to reproduce and complete the unfinished pioneering work on AS floc-formation mechanism. *Our results could shed more light on the molecular mechanism underlying formation of activated sludge flocs and bioflocculation.*

## Results

### Complete genome sequencing, annotation and comparative analysis

**Fig. 1:**
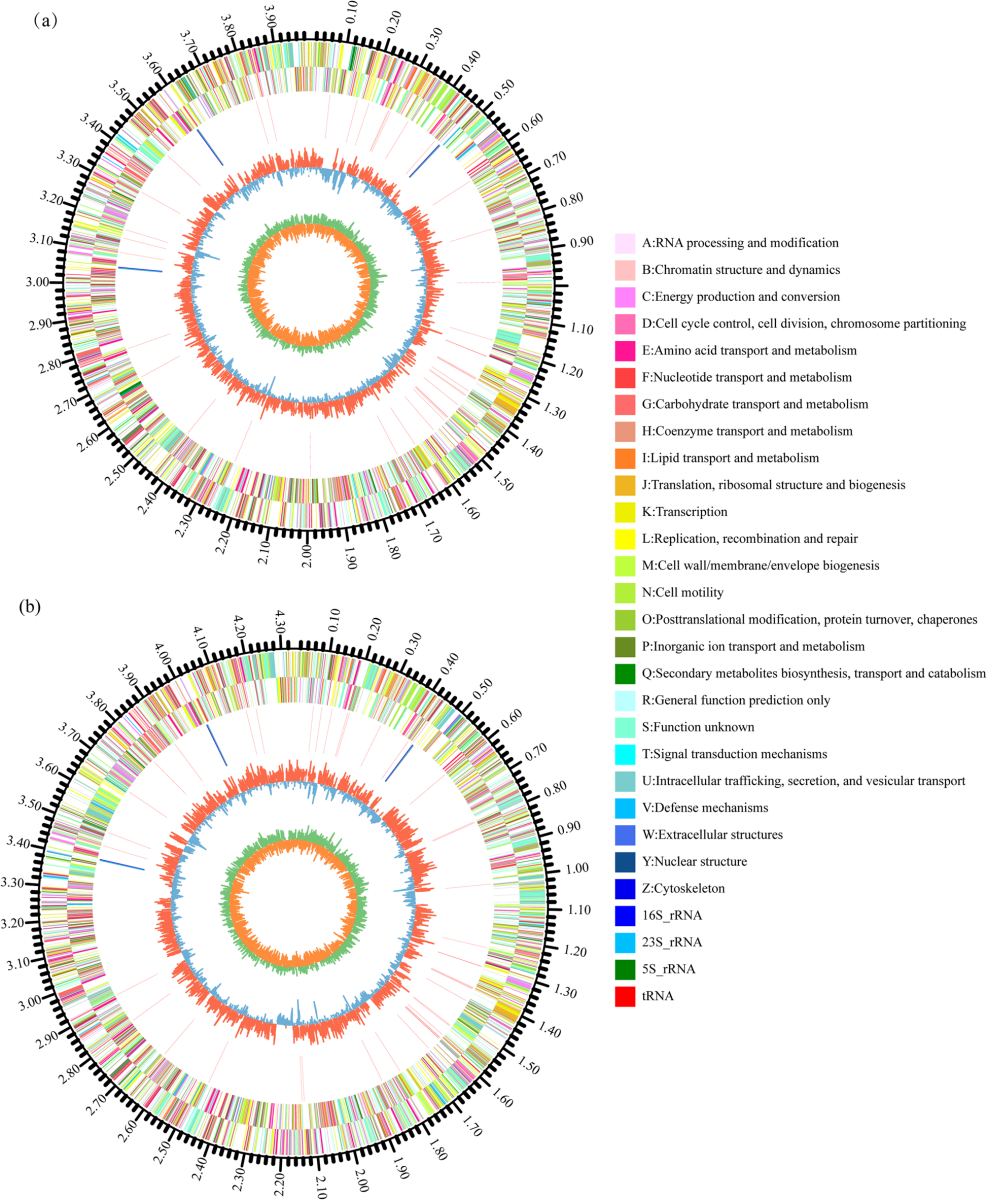
Circular genome maps of *Shinella zoogloeoides* ATCC 19623 (**a**) and XJ20 (**b**) from inside to outside indicate the following: Circle 1: G + C skew; green, GC skew>0; orange, GC skew<0; Circle 2: G + C content (median represents the above average content, the outer circle is greater than the average content (red), and the inner circle is less than the average content (blue)); Circle 3: rRNA and tRNA genes distribution represented in scaffold sequence; Circle 4 and 5: open reading frame (ORF) distribution, minus and plus strand, different colors represent the functional classification of COG with different CDS; Circle 6: indicator of genome size

The complete genome sequences of both *S. zoogloeoides* ATCC 19623 and XJ20 strains were sequenced by using both Illumina HiSeq and Pacific Biosciences platforms. The complete genome of *S. zoogloeoides* ATCC 19623 was 4,848,065 base pairs (bp) in size with the G + C content of 64.43%, containing 9 rRNAs operons, and 53 tRNAs. On the other hand, the genome of *S. zoogloeoides* XJ20 was 5,141,032 bp in size with the G + C content of 63.89%, and also contained 9 rRNAs operons, and 53 tRNAs. Interestingly, the *S. zoogloeoides* XJ20 strain encoded a higher number of protein-coding genes (5090) than that of *S. zoogloeoides* ATCC 19623 (4687), which was consistent with its larger genome size. Moreover, there were 6 plasmids in XJ20 and 4 plasmids in *S. zoogloeoides* ATCC 19623, respectively (Table 1). The single circular genome maps of the two *S. zoogloeoides* genomes were shown in Fig. 1. Using a Venn diagram of two strains, the majority of homologous gene groups and unique gene groups were identified. The unique gene groups accounted for 880 and 1194 gene groups in *S. zoogloeoides* ATCC 19623 and *S. zoogloeoides* XJ20, respectively (Supplemental Fig. 1). The average genomic nucleotide identity (ANI) between *S. zoogloeoides* XJ20 and *S. zoogloeoides* ATCC 19623 was 93.88%, which was lower than the species delineation thresholds (95–96%), suggesting that *S. zoogloeoides* XJ20 might not belong to the same species *sensu stricto* [23]. We conducted comparative genomics analysis of the two strains. These two chromosome genomes were aligned with Progressive Mauve using default parameters [24]. The genome synteny and collinearity between strain *S. zoogloeoides* ATCC 19623 and *S. zoogloeoides* XJ20 was high (Supplemental Fig. 2), though there were still some rearrangements and translocation. However, the large gene cluster for exopolysaccharide biosynthesis and secretion, PEP-CTERM proteins and the PrsK-PrsR two-component system regulating the expression of PEP-CTERM proteins, which are required for the floc-forming of *Z. resiniphila* MMB strain and *A. tertaricarbonis* RN12 strain [17–19], were not encoded in the genome of *S. zoogloeoides* ATCC 19623, *S. zoogloeoides* XJ20 and other sequenced *Shinella* strains. It was strongly suggested that the floc-forming mechanism in *Shinella* species was different from that of *Zoogloea* species, one of the predominant floc-forming bacteria in activated sludge.

**Table 1:** General characteristics of the *Shinella zoogloeoides* two strains genome

Characteristics	ATCC 19623	XJ20
Genome size (bp)	4,848,065	5,141,032
GC content (%)	64.43	63.89
Number of Subsystems^a^	380	384
Number of coding sequences	4788	5203
Number of genes	4687	5090
Gene total length (bp)	4,242,516	4,487,895
Gene density	0.97	0.99
Number of plasmids	4	6
Number of tRNAs	53	53
Number of rRNAS	9	9

^a^Subsystems represent the collection of functional roles that make up a metabolic pathway, a complex, or a class of proteins

### Identification of a bacterial cellulose synthesis (*bcs*) gene cluster required for floc formation

**Fig. 2:**
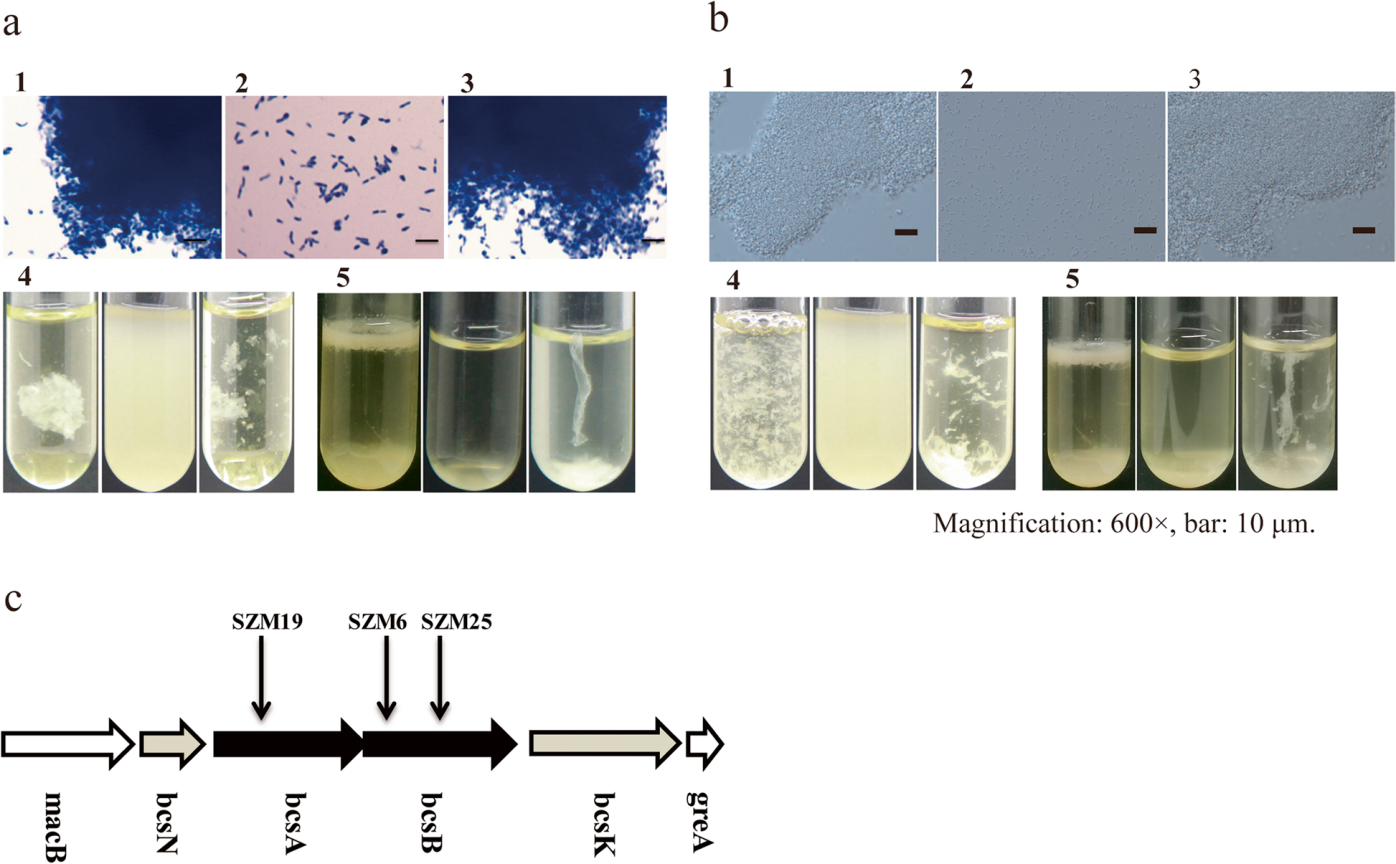
The genetic complementation analysis of the *bcsA* and *bcsB* genes in transposon mutant SZM19, SZM6 and SZM25. **a** The genetic complementation analysis of the *bcsA* in trans in the mutants SZM19. Plasmid-borne wild-type *bcsA* gene restored the phenotype of the SZM19 mutant to floc formation, similar to that of the wild-type strain carrying the empty vector. (1) Wild-type strain with the pBBR1MCS-2 empty vector, visualized under a microscope after staining with crystal violet solution. (2) SZM19 mutant with pBBR1MCS-2. (3) SZM19 mutant with pBBR1MCS-2-*bcsA* construct. (4) Photograph of agitated bacterial cultures (culture tubes contain bacteria from panels (1) to (3), from left to right). (5) Photograph of settled bacterial cultures. **b** The genetic complementation analysis of the *bcsB* in trans in mutants SZM6. (1) Wild-type strain with the pBBR1MCS-2 empty vector, visualized under a microscope. (2) SZM6 mutant with pBBR1MCS-2. (3) SZM6 mutant with pBBR1MCS-2-*bcsB* construct. (4) Photograph of agitated culture (culture tubes contain bacteria from panels (1) to (3), from left to right). (5) Photograph of settled bacterial cultures. **c** The cellulose biosynthesis gene cluster of *Shinella zoogloeodies* ATCC 19623 strain and the genes disrupted by insertions of the mariner transposon, mapped in the transposon mutants deficient for bacterial floc formation

Previously the *Tn5* transposon mutagenesis had been conducted on the floc-forming activated sludge bacterium *S. zoogloeoides* ATCC 19623 (still classified as *Z. ramigera* I-16-M at that time) strain to identify the genes required for biosynthesis of extracellular polysaccharides and floc formation [16]. A series of mutants deficient in exopolysaccharide biosynthesis were isolated and plasmids from a genomic library, containing at least 14 kilobases of common insert DNA, could restore the floc forming phenotype of the mutants after entering the cells via conjugation. However, the DNA sequence and relevant genes had not been determined due to the technical limitation at that time. In the present study, the transposon mutagenesis was conducted on the *S. zoogloeoides* ATCC 19623 strain by using *mariner* transposon as previously described [25]. Sixty-one floc-forming-deficient mutants had been isolated and the transposon insertional site had been mapped in nineteen mutants (Supplemental Table S1). As presented in Fig. 2 a5 and b5, these mutants do not form the flocs in the agitated liquid culture or the thick pellicle on the surface of the static liquid culture. Microscopic observation shows that the bacterial cultures of those mutants are usually composed of single cells and tiny cell aggregates (Fig. 2 a2, 2b2), but not large flocs as previously shown [17–19]. For example, one of the mutants has interrupted *bcsA* and the other two *bcsB*. Furthermore, the plasmid-borne *bcsA* gene (pBBR1MCS-2-*bcsA*) and *bcsB* gene (pBBR1MCS-2-*bcsB*) restored the floc formation phenotype to these two mutants in genetic complementation analyses (Fig. 2a, b). These mutants were mapped to a bacterial cellulose synthesis (*bcs*) gene cluster (locus tags K8M09_02915- K8M09_02940). This gene cluster was composed of *bcsN*, *bcsA*, *bcsB* and *bcsK* (Fig. 2c).

### The restriction map of *bcs* gene cluster-flanking region is highly similar to that of previously identified exopolysaccharide biosynthesis gene region

**Fig. 3:**
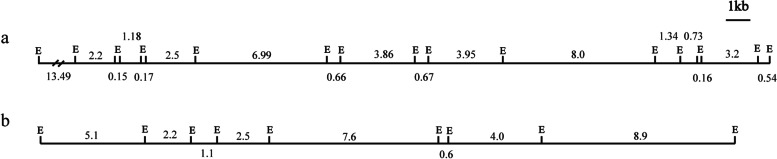
Comparison of the two *Eco*R I restriction maps. **a** *Eco*R I restriction maps of pPS25, pPS27, pPS30, pPS48, and the composite region identified from the complementing cosmids of gene library by Easson, Sinskey and Peoples [16]; **b** The digital restriction map of the identified *bcs* gene cluster-flanking region. Sizes of each fragment are shown in kilobases. Illustrator for Biological Sequences (IBS1.0.3) and Adobe Illustrator CS6 programs were used to draw the maps

We constructed a digital restriction map of the identified *bcs* gene cluster-flanking region, which was highly similar to that of the 32 kilobases exopolysaccharide biosynthesis gene region previously identified from the complementing cosmids of gene library by Easson, Sinskey and Peoples [16]. The counterpart of most of the *Eco*R I restriction fragments could be found in the digital restriction map and the measured size of these fragments were close to their real length, suggesting that the quality of the original restriction map was good (Fig. 3). The inconsistency between the two maps might be due to the technical limitation of restriction mapping. Some small restriction fragments could not be readily identified by the agarose gel electrophoresis and ethidium bromide staining methods and were missed in the original restriction map. Nevertheless, the resolution of agarose gel electrophoresis was not good enough to differentiate the bands containing two or more restriction fragments of similar sizes. These results indicated that we have largely reproduced and further confirmed the previous findings of Easson, Sinskey and Peoples [16].

### Cellulase supplement prevented the floc formation of *Shinella* strains but not *Z. resiniphila*

**Fig. 4:**
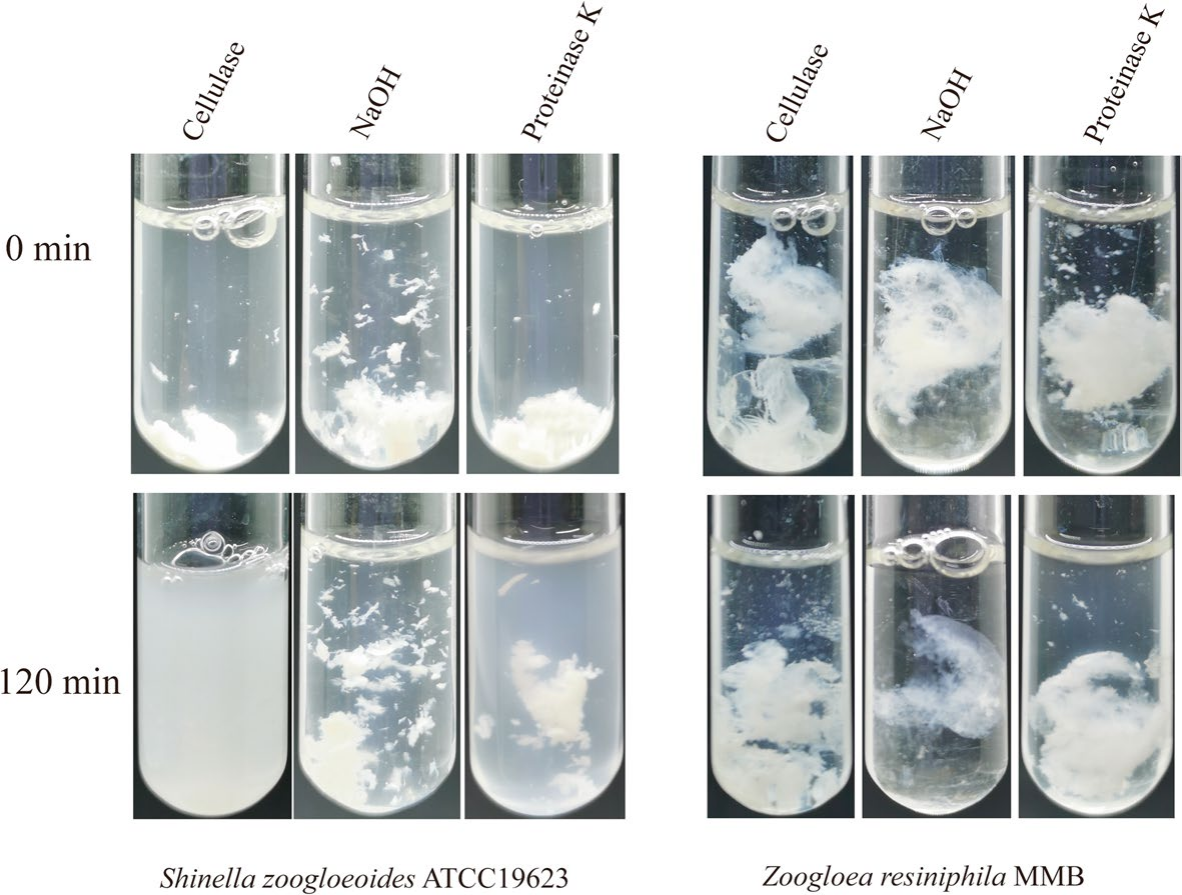
Treatment of *Shinella zoogloeoides* ATCC 19623 and *Zoogloea resiniphila* MMB cell flocs with cellulase, proteinase K and NaOH solution

Spontaneous flocculation of *Escherichia coli* cells could be induced by overexpression of the *bcsB* gene and those flocs were sensitive to proteinase K and NaOH, but not to cellulase, indicating that the main component of the flocs was proteinaceous [26]. In this study, we added the commercial cellulase, proteinase K or NaOH solution to the bacterial cultures of *Z. resiniphila* MMB and *S. zoogloeoides* ATCC 19623. Our results showed that the flocs of *Shinella* could be disrupted by addition of cellulase, while the floc formation of the *Z. resiniphila* MMB strain was not affected (Fig. 4). Both proteinase K and NaOH could not de-flocculate the flocs of *Shinella*, only *Z. resiniphila* MMB exhibited slight sensitivity to NaOH solution. These results further verified that the extracellular polymers required for floc formation of *Shinella* were the cellulose materials vulnerable to cellulase degradation.

### Characterization of bacterial exopolymers

**Fig. 5:**
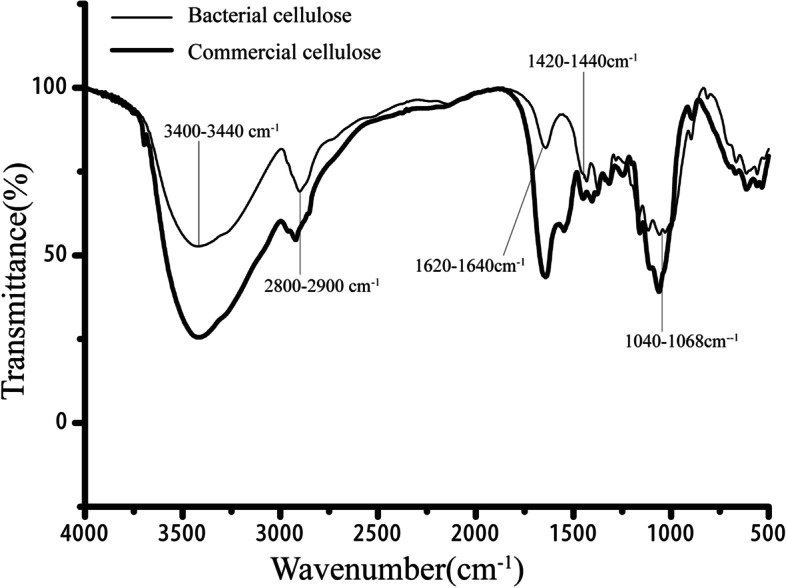
FTIR spectra of bacteria cellulose from *Shinella zoogloeoides* ATCC 19623 and commercial cellulose. NICOLET 5700 FTIR Spectrometer was used with diamond ATR spectrophotometer for 24 scans in the range of 4000–400 cm^−1^ with a resolution of 4 cm^−1^. Bacteria cellulose extracted from *Shinella zoogloeoides* ATCC 19623, commercial cellulose purchased from Sangon Biotech, Shanghai, China

The involvement of cellulose in the formation of flocs has been confirmed by molecular genetics. According to the extraction methods of cellulose from other strains, FTIR analysis was used to prove that the substance extracted from *S. zoogloeoides* ATCC 19623 was cellulose. The FTIR spectra of all the cellulose could provide the information about their functional groups and the state of bonds in their structure (Fig. 5). The FTIR spectrum of bacterial cellulose sample was highly comparable with that of the commercial cellulose product used as the control. The hydroxyl groups were observed at ~3400–3440 cm^−1^, which indicated (−OH) stretching vibrations. The broader peak bands indicate intermolecular hydrogen bonding in the cellulose molecule, and also these hydroxyl groups are responsible for facilitating substitution in the molecule, consistent with the previously reported results [27]. The peak at ~2800–2900 cm^−1^ represents symmetric (−CH) stretching vibrations indicating the presence of methyl/methylene functional group. The peak at ~1620–1640 cm^−1^ reveals a water molecule associated with cellulose (absorbed H_2_O) bending vibrations. The carbonyl groups (C=O) are observed at the ~1420–1440 cm^−1^. The peak at~1040–1068 cm^−1^ indicates the presence of (C–O) stretching vibration in the polymer. These significant peaks further confirmed that the extracted sample was a cellulose polymer.

## Discussion

Based on the core community of activated sludge [28] and our own isolation of AS bacteria, many predominate AS bacteria exhibited floc-forming phenotypes, such as small flocs and less strengths, similar to those of *S. zoogloeoides*. Those key AS bacteria such as *Acidovorax*, *Comamonas*, *Diaphorobacter* and *Hydrogenophaga* actually lack the PEP-CTERM genes and EPS biosynthesis genes previously described for *Z. resiniphila*, instead, they encode cellulose biosynthesis pathway similar to that of *Shinella* described in this study. The mechanism underlying the floc formation of *Shinella* is different from that of *Zoogloea* and other predominant activated sludge bacteria in which both the PEP-CTERM proteins and extracellular polysaccharides are required for floc formation [17–19].

The *Shinella* genus is phylogenetically close to the *Agrobacterium* and *Rhizobium* genera of *Hyphomicrobiales*, and *S. zoogloeodies* ATCC 19623 exhibits a floc-forming phenotype, which requires bacterial cellulose synthesis as shown previously [16] and in this study. The comparative genomic analysis showed that *S. zoogloeodies* ATCC 19623 was highly collinear to *S. zoogloeodies* XJ20 isolated from Xinjiang (Supplemental Fig. 2). Sixty-one floc-forming-deficient mutants had been isolated and the transposon insertional site has been mapped in nineteen mutants in the *S. zoogloeodies* ATCC 19623 strain. Genetic complementation was conducted to confirm whether the identified genes are required for floc-formation and the insertion site of three mutants was mapped to a bacterial cellulose synthesis (*bcs*) gene cluster. This gene cluster was composed of *bcsN*, *bcsA*, *bcsB* and *bcsK* (Fig. 2), which could be found in other bacteria, like *Agrobacterium fabrum* C58 and *Azospirillum lipoferum* 4B [29]. Omadjela *et al*. reported that BcsA and BcsB are necessary and sufficient for the formation of the polysaccharide chain *in vitro* in most studied bacteria [30]. The protein encoded by *bcsA* contains the catalytically active subunit with a PilZ domain, which is responsive to *c-di*-GMP. BcsB is located in the periplasm and is anchored in the membrane by a single transmembrane helix [31]. The functions of BcsK are probably the same as those of AlgK and the N-terminal part of BcsC, i.e., interacting with the peptidoglycan and organizing the entire cellulose secretion complex [29]. The functions of BcsN remain unclear. Our results clearly demonstrated that bacterial cellulose biosynthesis was required for floc formation of *S. zoogloeoides* ATCC 19623 strain. The FTIR analysis showed the sample extracted from *S. zoogloeoides* ATCC 19623 was cellulose polymer (Fig. 5). Biosynthesis and regulatory pathways of cellulose are also highly conserved among these bacteria of *Hyphomicrobiales* [32]. *Shinella* has been frequently found in the activated sludge samples, but it may not be one of the predominant genera because the *Shinella* genus is not among the 28 taxa of global core community of activated sludge [28]. On the other hand, the floc-forming bacteria including *Zoogloea, Thauera* and *Dechloromonas* are among the core community and encode the exopolysaccharide biosynthesis gene clusters and PEP-CTERM domain containing proteins. The fluorescent probe complementary to the 16S ribosomal RNAs of the *S. zoogloeoides* ATCC 19623 strain bound to only a few cells of activated sludge samples [33]. But this kind of small and unstable flocs, which as minor players of high species diversity in AS, still facilitates integration of these bacteria into AS flocs.

As compared to the highly diverse exopolysaccharides, cellulose is more degradable and vulnerable because many microbes synthesize and secrete cellulase. Our results have also shown that the flocs of *Shinella* could be disrupted by addition of cellulase (Fig. 4). Bacteria cellulose biosynthesis is regulated by the expression of *bcs* genes, which appear to be expressed in response to the second messenger cyclic-*di*-GMP [34]. The c-*di*-GMP is synthesized by diguanylate cyclases (DGCs) that carry GGDEF domains and degraded by phosphodiesterases (PDEs) that carry either EAL or HD-GYP domains, all these domains are essential for enzymatic activity [35]. Indeed, even though the GGDEF and EAL domain-containing proteins have opposing activities, these two domains are often found coupled in the same proteins, which are referred to as hybrid proteins because they carry both domains [36]. We found eight hybrid proteins containing GGDEF and EAL domain, six stand-alone c-*di*-GMP PDEs and four stand-alone DGCs are present in the genome. These multiple genes together control the intracellular *c-di*-GMP levels. Easson *et al*. described spontaneous mutations and genetic instability of the strain *Shinella* in the region encoding the cellulose biosynthetic cluster [16]. Genome instability can be mediated by genetic elements, homologous and illegitimate recombination. A number of mobile elements (including insertion sequences, miniature inverted-repeat transposable elements, repetitive extragenic palindromic sequences and bacterial interspersed mosaic elements, transposons, transposable bacteriophages and genomic islands), introns and integrons play a role in genomic instability [37]. The number of gene islands, prophage and insertion sequences in the genome of *S. zoogloeoides* ATCC 19623 was 20, 4, 11, respectively. It remains unknown whether other genes are required for the floc formation of *S. zoogloeoides* ATCC 19623, in addition to the *bcsA* and *bcsB* genes involved in biosynthesis of extracellular cellulose polymers. *Shinella* strains may serve as a good bioflocculant of wide-range of applications.

## Conclusions

In this study we further explored the mechanism underlying the floc formation of *S. zoogloeoides* ATCC 19623 (previously *Zoogloea ramigera* I-16-M) and related activated sludge bacteria, and sequenced and annotated the complete genome of *S. zoogloeoides* ATCC 19623 and XJ20 strains. The comparative genomic analysis showed that the chromosomes of *S. zoogloeoides* ATCC 19623 and XJ20 strain were highly collinear, although they were isolated from activated sludge of United States and China, respectively. Our results clearly demonstrate that bacterial cellulose biosynthesis gene *bcsA* and *bcsB* are required for floc formation of *S. zoogloeoides* ATCC 19623 and the flocs could be disrupted by addition of cellulase. This mechanism may account for the smaller and less stable flocs formed by highly diverse bacteria in activated sludge. These results indicate that we have largely reproduced and completed the unfinished pioneering research on the genes required for floc-forming in *S. zoogloeoides* ATCC 19623 by Easson, Sinskey and Peoples three decades ago.

## Methods

### Bacterial strains, plasmids, and culture conditions

The bacterial strains and plasmids used in this study are listed in Supplemental Table S2. The *S. zoogloeoides* ATCC 19623 strain was obtained from China Center for Type Culture Collection (CCTCC) and the XJ20 strain was isolated from the activated sludge of Municipal Sewage Treatment Plant, Xinjiang Uygur Autonomous Region, China, collected by Yaqi Liu in September, 2019. Bacterial strains were cultured in the Luria-Bertani broth (5 g/L yeast extract, 10 g/L tryptone, 10 g/L sodium chloride (NaCl, pH 7.0)/plates (supplemented with 15–30 μg/ml of gentamycin or 50 μg/ml of kanamycin and 50 μg/ml of diaminopimelic acid, when necessary), and *Zoogloea* medium (ZM) [19] at 28 °C.

### Genome sequencing, annotation and comparative genomic analysis

Two complete genomes of *S. zoogloeoides* were sequenced using a combination of PacBio RS and Illumina sequencing platforms. The Illumina data was used to evaluate the complexity of the genome and correct the PacBio long reads. Firstly, we used the SOAP de novo version 2.21 package to do genome assembly with multiple-Kmer parameters (21–41) and got the optimal results of the assembly. Secondly unicycler was used to assemble the PacBio corrected long reads. Finally, GapCloser software was subsequently applied to fill up the remaining local inner gaps and correct the single base polymorphism for the final assembly results. The complete circle of the genome was drawn with Circos v0.64. Genome annotation was performed using RAST (Rapid Annotation using Subsystem Technology). The rRNA genes were predicted by RNAmmer [38], and tRNAscan-SE [39] was used to identify the tRNA genes. The interactive web-based software system GC-Profile [40] was used to investigate the genomic GC content. The genomes alignment was performed by Progressive Mauve [24]. Nucleotide and protein sequences were retrieved from NCBI database by using BLAST searches at the National Center for Biotechnology Information (http://www.ncbi.nlm.nih.gov/BLAST). Homologous gene analysis was performed using OrthoMCL program. The relationship between sequenced strains was determined by Average Nucleotid Identity (ANI) calculation with web tool ANI calculator [41] (https://www.ezbiocloud.net/tools/ani).

### Transposon mutagenesis and genetic complementation

The *mariner* transposon mutant libraries were generated and screened as previously described [17, 42, 43], with *Escherichia coli* WM3064 strains carrying the transposon delivery suicide plasmids pFAC (courtesy by Dr. John Mekalanos, Harvard Medical School) as the donor and the *S. zoogloeoides* ATCC 19623 strain as the recipient for biparental conjugation [18]. The transposon insertion site in each mutant was mapped as previously described [17]. For genetic complementation analyses, the target genes were PCR amplified and cloned into the pBBR1MCS-2 [44]. The primers for PCR reactions are listed in the Supplemental Table S2. The resultant constructs and empty vector were transferred into the *S. zoogloeoides* wild type strain and mutant strains via conjugation using WM3064 as a donor strain.

### Assays of flocs sensitivity to enzyme treatment

Floc formation was studied in the ZM medium [19]. *Z. resiniphila* MMB has been used as negative control. Each tube contained 3 ml of medium, incubation was carried out on a rotary shaker at 28 °C for 36 h After the flocs were settled and washed three times with water in the same test tube by decantation. The collected flocs were treated with 2 mg/ml cellulase (*Trichoderma reesei* ATCC 26921, Sangon Biotech, Shanghai, China) in 2.5 mM 2-morpholinoethanesulfonic acid buffer (pH 5.5), 2 mg/ml proteinase K (*Tritirachium album limber*, Sangon Biotech, Shanghai, China) in 50 mM Tris buffer (pH 8.0), or 0.5 M NaOH solution at 37 °C for 2 h [28].

### Extracellular polymer extraction, purification and spectrometry analyses

Aliquots of 15 ml bacteria culture were inoculated into a flask containing 1.5 L of ZM medium. Later the conical flasks were left for static incubations at 28 °C. Cells were harvested by centrifuge after a week of incubation and cell pellets were washed with deionized water to remove the residual medium. The cell pellets were then boiled in 1% NaOH at 80 °C for 90 min to dissolve bacterial cell mass and rinsed with deionized water until the filtrate reached neutral pH value. The purified sample was then dried by freeze-drying. Dried polymers were analyzed for both chemical and structural features with commercial cellulose as a reference [43]. FTIR spectral analyses were performed to ascertain the similarity between the functional groups of the sample and the reference molecule.

## Supplementary Information

**Additional file 1: Supplemental Figure 1.** Venn diagram showing the number of genes shared by both the strains and unique genes in *Shinella zoogloeoides* ATCC19623 and XJ20 strain. **Supplemental Figure 2.** Nucleotide-based alignment of the choromosome genomes from the two strains. Homologous blocks are shown as identically colored regions and linked across the genomes. Regions that are inverted relative to ATCC19623 are shown below the central axis of each sequence. These two chromosome genomes were aligned with Progressive Mauve using default parameters. **Supplemental Table S1.** Transposon mutants of *Shinella zoogloeoides* ATCC 19623 defective in floc formation, mapped insertional sites and genetic complementation ATCC19623. **Supplemental Table S2.** Bacterial strains, plasmids and primers used in this study.

## Data Availability

The complete genome sequencing datasets of *Shinella zoogloeoides* ATCC 19623 and *Shinella zoogloeoides* XJ20 used in this study were deposited in GenBank under accession CP086610 for ATCC 19623 chromosome, CP086611 - CP086614 for ATCC 19623 plasmids, CP093528 for XJ20 chromosome, CP093529 - CP093534 for XJ20 plasmids.
