# Supplementary material for: Cellulose-mediated floc formation by the activated sludge bacterium Shinella zoogloeoides ATCC 19623
Source: BMC Microbiol. 2022 Apr 15;22:104. doi: 10.1186/s12866-022-02516-y (PMC9012009; doi:10.1186/s12866-022-02516-y)
Supplement: Supplementary file 1 — Additional file 1: Supplemental Figure 1. Venn diagram showing the number of genes shared by both the strains and unique genes in Shinella zoogloeoides ATCC19623 and XJ20 strain. Supplemental Figure 2. Nucleotide-based alignment of the choromosome genomes from the two strains. Homologous blocks are shown as identically colored regions and linked across the genomes. Regions that are inverted relative to ATCC19623 are shown below the central axis of each sequence. These two chromosome genomes were aligned with Progressive Mauve using default parameters. Supplemental Table S1. Transposon mutants of Shinella zoogloeoides ATCC 19623 defective in floc formation, mapped insertional sites and genetic complementation ATCC19623. Supplemental Table S2. Bacterial strains, plasmids and primers used in this study. [file 12866_2022_2516_MOESM1_ESM.docx]

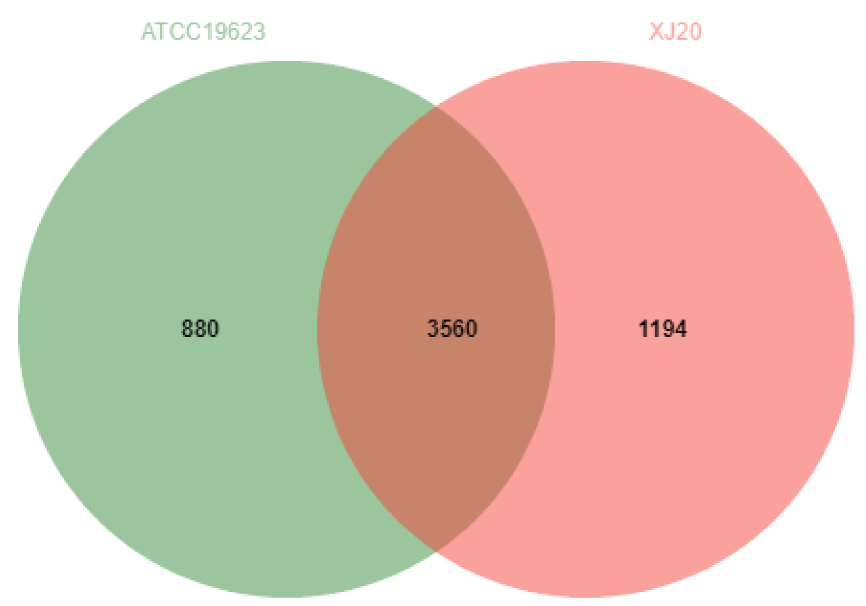


**Supplemental Figure 1.** Venn diagram showing the number of genes shared by both the strains and unique genes in *Shinella zoogloeoides* ATCC19623 and XJ20 strain.


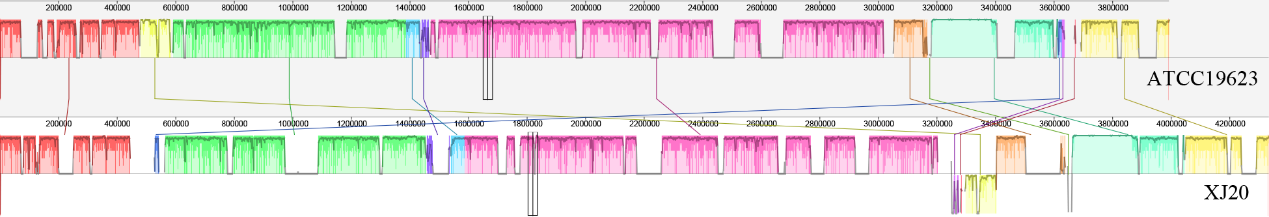


**Supplemental Figure 2**. Nucleotide-based alignment of the choromosome genomes from the two strains. Homologous blocks are shown as identically colored regions and linked across the genomes. Regions that are inverted relative to ATCC19623 are shown below the central axis of each sequence. These two chromosome genomes were aligned with Progressive Mauve using default parameters.

**Supplemental Table S1** Transposon mutants of *Shinella zoogloeoides* ATCC 19623 defective in floc formation, mapped insertional sites and genetic complementation ATCC19623.

| Gene product | Mutants | Floc formation | Insertion site | Genetic complementation |
| --- | --- | --- | --- | --- |
| Oxidoreductase | SZM17 | defective | TGGTGAACCT**TA**ACAGGTT | Not yet |
| Plasmid replication protein RepA | SZM11 | defective | ATTACGCGAG**TA**ACAGGTT | Not yet |
| BcsA | SZM19 | defective | GCCGCTCTTC**TA**ACAGGTTG | Yes |
| Mobile element protein | SZM5 | defective | GTCCATCGAGGACA**TA**ACAG | Not yet |
| HlyD family secretion protein | SZM2 | defective | AGACCCGCCGCCA**TA**ACAGG | Not yet |
| Beta-galactosidase, LacA family | SZM21 | defective | TCGACGCCATGCGCA**TA**ACAG | Not yet |
| Decarboxylase family protein | SZM27 | defective | GCGCCGAACCCC**TA**ACAGGTTG | Not yet |
| hypothetical protein | SZM48 | defective | TCAAGGGGACCA**TA**ACAGGT | Not yet |
| BcsB | SZM6 | defective | GTCTGGCCGT**TA**ACAGGTTG | Yes |
|  | SZM25 |  | TCTGGCCGTTA**TA**ACAGGTT |  |
| ClpB protein | SZM43 | defective | TACGACCCGGTC**TA**ACAGGTT | Not yet |
| LuxR family transcriptional regulator | SZM54 | defective | CGACATGGAAGGC**TA**ACAGGTT | Not yet |
| UbiD family decarboxylase | SZM41 | defective | CGGCAATCTCAGC**TA**ACAGGTT | Not yet |
| Flagellum biosynthesis repressor protein FlbT | SZM36 | defective | GGGCAATGAATTC**TA**ACAGGTT | Not yet |
| Flagellar synthesis regulatory protein FlaF | SZM37 | defective | GCGCGTCGCGGCCTCA**TA**ACAGG | Not yet |
| Cytochrome c heme lyase subunit CcmL | SZM24 | defective | TGCCGCAGACAGTGTGCTG**TA**AC | Not yet |
| Alpha-aminoadipate aminotransferase | SZM22 | defective | GCCGCTCTCGATAAG**TA**ACAGGT | Not yet |
| Aldose 1-epimerase | SZM13 | defective | GGGAGTGGGCGAGA**TA**ACAGGTT | Not yet |
| glutaryl 7-ACA acylase precursor | SZM32 | defective | TCTCCAAAACTCGCC**TA**ACAGGTT | Not yet |

**Supplemental Table S2** Bacterial strains, plasmids and primers used in this study

| Strains | | Description | | | Source or reference | |
| --- | --- | --- | --- | --- | --- | --- |
| *Escherichia coli* WM3064 | | *thrB1004 pro thirpsLhsdS lacZDM15 RP4-1360 (araBAD)567dapA1341::[ermpir(wt)]* | | | W. Metcalf | |
| *Zoogloea. resiniphila* MMB | | Activated sludge bacterium, floc formation | | | This study | |
| *Shinella zoogloeoides* ATCC19623 | | The type strain of *Shinella zoogloeoides*, floc formation | | | China Center for Type Culture Collection (CCTCC) | |
| *Shinella* *zoogloeoides* XJ20 | | Activated sludge bacterium, floc formation | | | This study | |
| SZM19 | | mariner transposon insertion in *bcsA* gene | | | This study | |
| SZM6 | | *mariner* transposon insertion in *bcsB* gene | | | This study | |
| SZM25 | | *mariner* transposon insertion in *bcsB* gene | | | This study | |
|  | | | | | | |
| Plasmids | | | | | |  |
| pBBR1MCS-2 | | | Broad-host-range cloning vector, Kan^r^ | (1) | |  |
| pFAC | | | *Mariner* transposon delivery plasmid R6K, Gmr | (2) | |  |
| pBBR1MCS2-*bcsA* | | | The *bcsA* gene cloned in pBBR1MCS-2 | This study | |  |
| pBBR1MCS2-*bcsB* | | | The *bcsB* gene cloned in pBBR1MCS-2 | This study | |  |
| primers | Oligonucleotide sequence (5’-3’) | | | | | |
| Gm-5OUT | 5’- TGCGTTCGGTCAAGGTTCTG -3’ | | | | | |
| Gm-3OUT | 5’- GGGCATACGGGAAGAAGTGA -3’ | | | | | |
| bcsA-F | 5’-GCTCTGCGACATGTGATTGAA-3’ | | | | | |
| bcsA-R | 5’-GTGTCTTGGCTTTCTGTTCC-3’ | | | | | |
| bcsB-F | 5’-CAGGTCTCGCGGCGCTACAAT-3’ | | | | | |
| bcsB-R | 5’-CGATAGGGTTAACGGACGGTA-3’ | | | | | |

1. Kovach ME, Phillips RW, Elzer PH, Roop RM 2nd, Peterson KM. pBBR1MCS: a broad-host-range cloning vector. Biotechniques. 1994;16(5):800-802.

2. Wong SM, Mekalanos JJ. Genetic footprinting with mariner-based transposition in Pseudomonas aeruginosa. Proc Natl Acad Sci U S A. 2000;97(18):10191-10196.
